# A 3D-Printed Offline Nano-ESI Source for Bruker MS Instruments

**DOI:** 10.1021/jasms.3c00214

**Published:** 2023-08-21

**Authors:** Michael Götze, Lukasz Polewski, Leïla Bechtella, Kevin Pagel

**Affiliations:** Institut für Chemie und Biochemie, Freie Universität Berlin, 14195 Berlin, Germany; Fritz-Haber-Institut der Max-Planck-Gesellschaft, 14195 Berlin, Germany

## Abstract

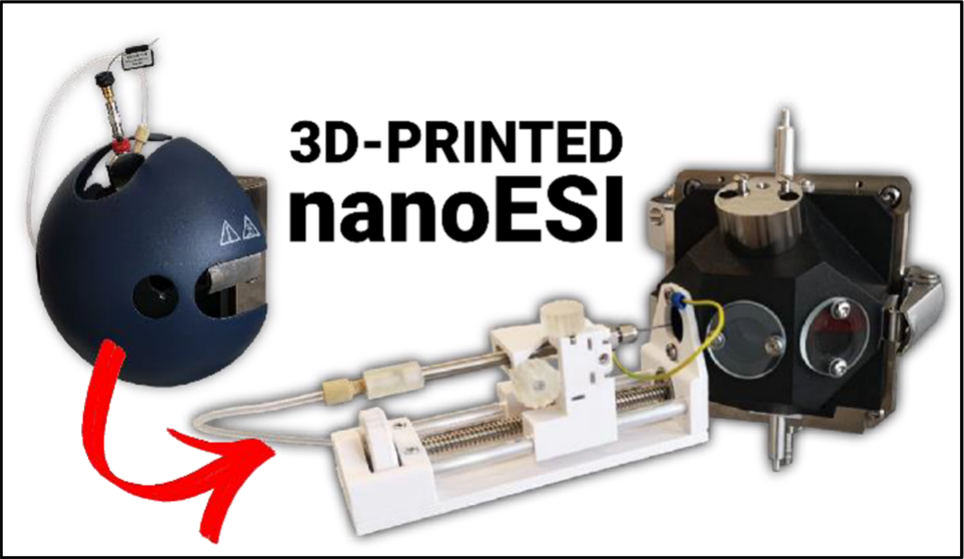

Nanoelectrospray ionization (nano-ESI) is a highly efficient and a widely used technique for the ionization of minute amounts of analyte. Offline nano-ESI sources are convenient for the direct infusion of complex mixtures that suffer from high matrix content and are crucial for the native mass spectrometric analysis of proteins. For Bruker instruments, no such source is readily available. Here we close this gap and present a 3D-printable nano-ESI source for Bruker instruments, which can be assembled by anyone with access to 3D printers. The source can be fitted to any Bruker mass spectrometer with an ionBooster ESI source and only requires minor, reversible changes to the original Bruker hardware. The general utility was demonstrated by recording high-resolution MS spectra of small molecules, intact proteins, as well as complex biological samples in negative and positive ion mode on two different Bruker instruments.

## Introduction

Nanoelectrospray ionization (nano-ESI)^1^ provides multiple advantages compared to normal flow electrospray ionization (ESI).^2^ During ESI, a liquid sample is dispersed into charged droplets as the result of an electric field from the emitter tip to the entrance of the mass spectrometer. Subsequently, the droplet size decreases due to solvent evaporation until their size reaches the Rayleigh limit followed by a series of Coulomb fissions until quasi-molecular ions are released following a complex mechanism.^3^ For nano-ESI the orifice of the emitter is reduced from 100 μm to a few microns^1^ or even submicron diameters.^4^ This significantly reduces the flow rate and leads to a much lower sample consumption. Due to these obvious advantages, online coupling of nanoflow HPLC and high-resolution mass spectrometry became standard in many mass spectrometry-based analytical workflows. However, certain complex mixtures and native proteins strongly benefit from direct infusion nanoelectrospray ionization. Beyond the straightforward determination of the molecular masses, this offline nano-ESI can be particularly useful to determine the stoichiometry of macromolecular complexes^5^ or the affinity of ligands bound to proteins.^6,7^

In its simplest implementation, a metal-coated glass emitter with a micron-sized orifice is filled with a few microliters of sample and placed in front of the inlet of a mass spectrometer under a static electric field capable of producing a stable spray. Implementations for different mass spectrometer types are commercially available. For Bruker instruments, a nano-ESI source for hyphenation to nanoflow HPLCs is available. This CaptiveSpray nano-ESI source can be connected to a nanoflow pump with an autosampler or a microliter syringe pump to analyze comparable sample amounts at the risk of cross-contamination or clogging the CaptiveSpray emitter. No add-on is currently available that would enable glass emitter-based offline nano-ESI on any of the Bruker mass spectrometers. To close this gap, we devised a simple, 3D-printed modification of an existing source block, the ionBooster ESI source, that allows nano-ESI from metal-coated glass emitters.

The modifications to the original source are fully reversible and enable tool-free switching between conventional ESI and nano-ESI within a few minutes. Detailed plans and instructions for the modification of the Bruker mass spectrometers are publicly available on the open platform Printables.com with the title “*NanoESI Source for Bruker*”.

## Results

**Figure 1:**
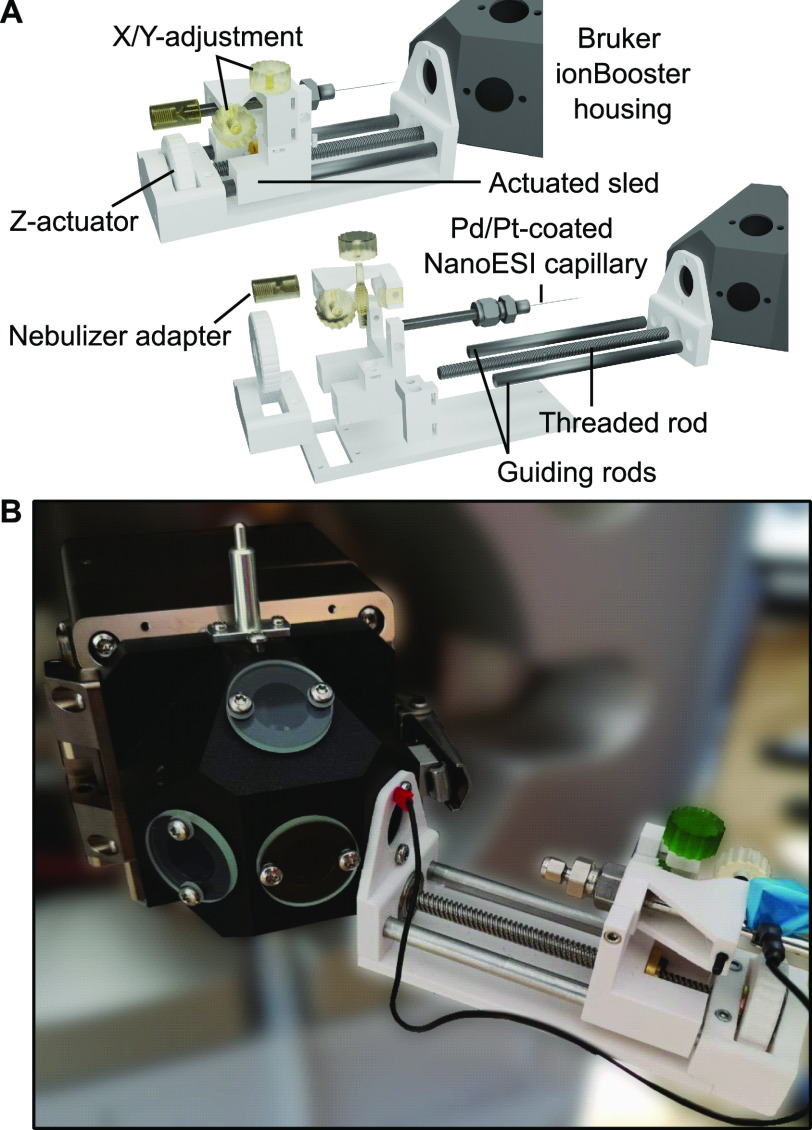
3D-printed offline nano-ESI source for Bruker instruments. (A) Model of the source adapter, which can be fitted to Bruker ionBooster source blocks. (B) Setup of the source on a Bruker timsTOF Pro. The emitter holder is connected to ground via the ionBooster housing.

To enable offline nanoelectrospray ionization on Bruker mass spectrometers such as the timsTOF, a nano-ESI emitter needs to be placed close to the entrance of the instrument and electrically connected to provide a stable voltage for the ionization process. This can be achieved through metal coating or inserting a wire into a glass emitter. Regardless of the intended ion polarity, the standard ESI-emitter in the Bruker ionBooster ESI source is held at ground potential by direct contact to the metal housing of the ion source. Nano-ESI emitters, therefore, simply need a contact to the instrument housing. The ionBooster source features three windows that can be used to observe the ESI spray. Any of these three windows provides access to the transfer capillary entrance. We devised a simple mechanism to guide an emitter close to the entrance capillary through one of these windows (Figure 1). The device (dimensions 55 × 75 × 155 mm) features a manually actuated sled carrying an emitter adapter (Swagelok), which can be rotated around a fixed point to position the emitter tip in front of the entrance capillary in the *x*- and *y*-dimensions and is connected to the source block by a ground wire to one of the mounting screws. Parts for the device were 3D-printed either from a PETG filament (poly(ethylene terephthalate glycol)) using a filament-based printer or from a UV-curable resin using a stereolithography printer. Nonprinted parts, e.g., rods and screws, can easily be sourced.

The resulting spray is stable without additional heating and does not require a nebulizer gas flow around the emitter. Instead, the nebulizer gas is utilized to pressurize the sample in the nano-ESI emitter and aid transport of the analyte solution in the tip. To achieve a stable spray, 1.2–1.8 kV emitter voltage, 500 V end plate offset, and backing pressure of 0.5–1.5 bar were applied.

**Figure 2:**
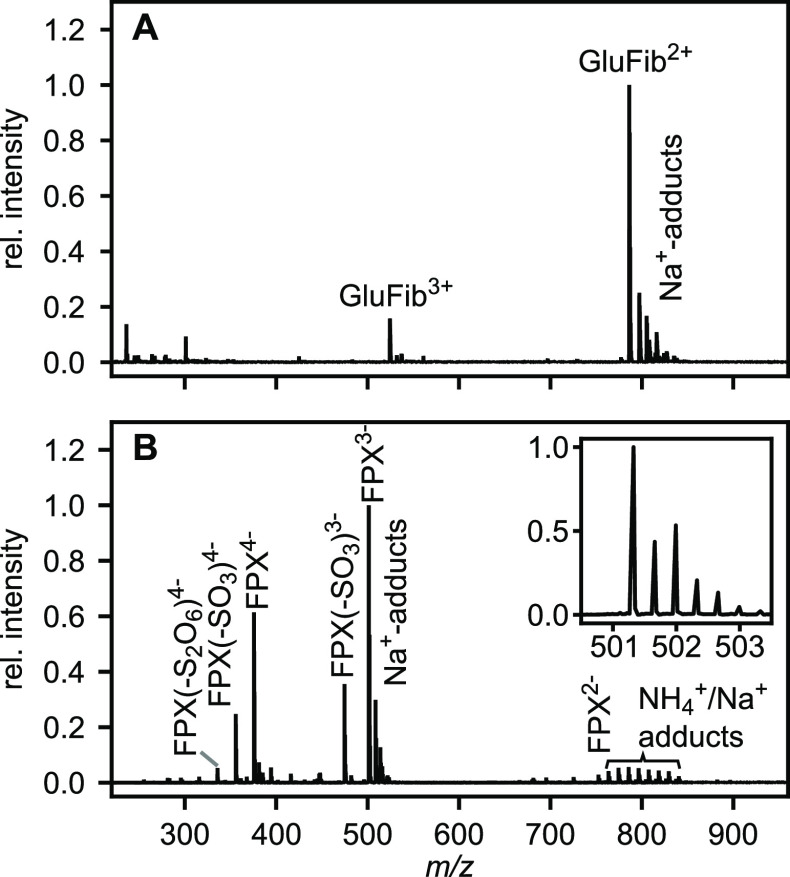
Mass spectrometric analysis of small molecules using nano-ESI. (A) Human fibrinopeptide B [Glu1] (EGVNDNEE-GFFSAR—1569.66 Da) was analyzed using a Bruker amaZon speed ETD mass spectrometer in positive mode. Doubly and triply charged species were observed. (B) Fondaparinux (FPX or Arixtra—1506.95 Da) was analyzed in negative ionization mode using a Bruker timsTOF Pro. Sodium adducts and loss of sulfate could be observed in multiple charge states. The inset shows the typical, expected isotopic distribution introduced by the eight sulfur atoms in FPX for the triply charged ion (*m*/*z* 501.32).

To initially test the utility of the nano-ESI source, pure, low molecular weight standards were used. The human fibrinogen peptide B [Glu1] or short GluFib^8^ served as a peptide standard and was observed in positive mode with +2 and +3 charges, as well as sodium adducts using the Bruker amaZon speed ETD instrument (Figure 2A).

The usually more delicate negative ionization mode was tested using a Bruker timsTOF Pro instrument and Fondaparinux (trade name Arixtra, short FPX) as a standard. FPX is a synthetic pentasaccharide, which is used clinically as an anticoagulent heparin mimic.^9,10^ In total, FPX contains eight sulfate groups (three *N*-sulfations and five *O*-sulfations). These glycan modifications are easily lost during ionization or activation similarly to other sulfated glycosaminoglycans.^11^ Intact FPX with −2 to −4 charges (Figure 2B) was detected. In addition, FPX ions with up to two sulfate losses were observed. However, these ions were much less abundant than the signal for intact FPX, which highlights the softness of the ionization.

**Figure 3:**
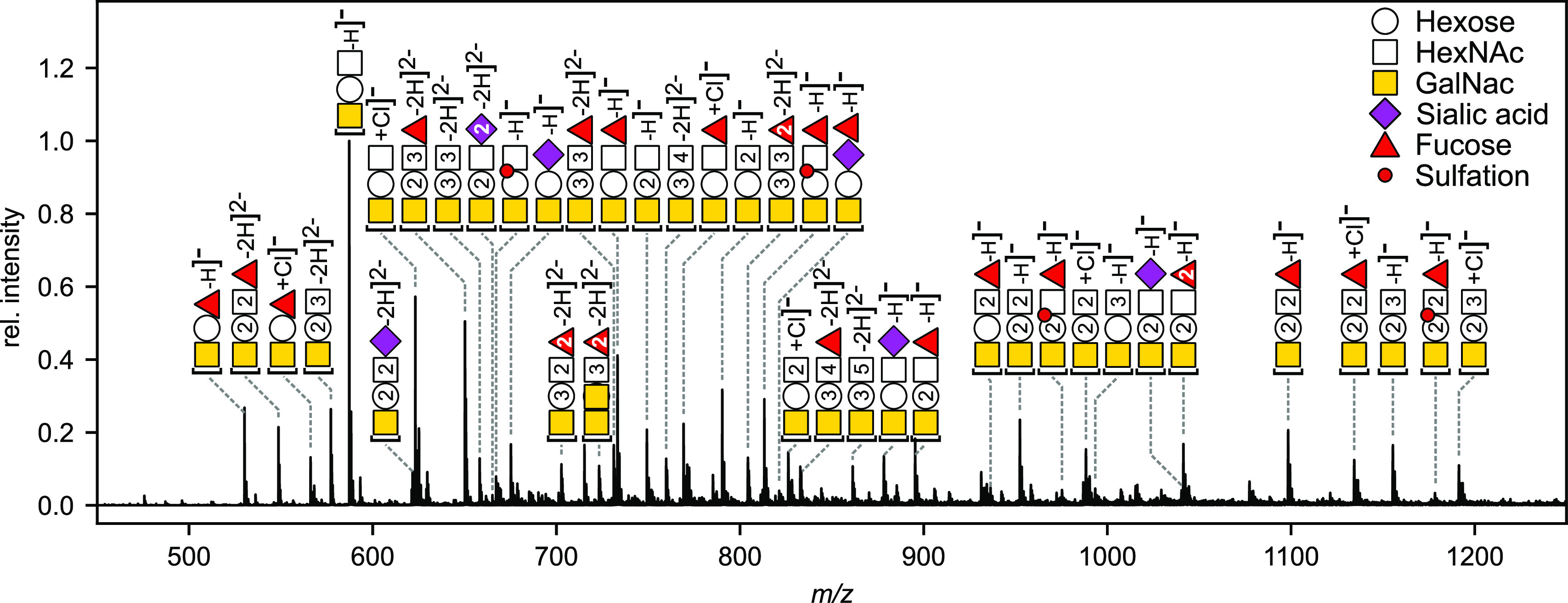
Mass spectrometric analysis of porcine gastric mucin (PGM) *O*-glycans. Multiple species occurred as singly and doubly charged ions arising from either proton loss or chloride adduct formation. The composition of each compound is shown using the Symbol Nomenclature For Glycans (SNFG),^12^ where the number of building blocks is noted inside the representation. The assigned compositions are listed in Table S1.

A more complex sample containing a mixture of *O*-glycans was generated from porcine gastric mucin (PGM) and analyzed using a Bruker timsTOF Pro in negative ionization mode (Figure 3.). *O*-Glycans were detected in −1 or −2 charge states, as deprotonated ions or chloride adducts. The major ions correspond to 32 glycan compositions ranging from tri- to octasaccharides with *m*/*z* values between 385.2 and 1724.7 Da (Table S1). The observed glycan distribution detected is very consistent with previous analyses also obtained by nano-ESI infusion using a Waters Synapt.^13^

**Figure 4:**
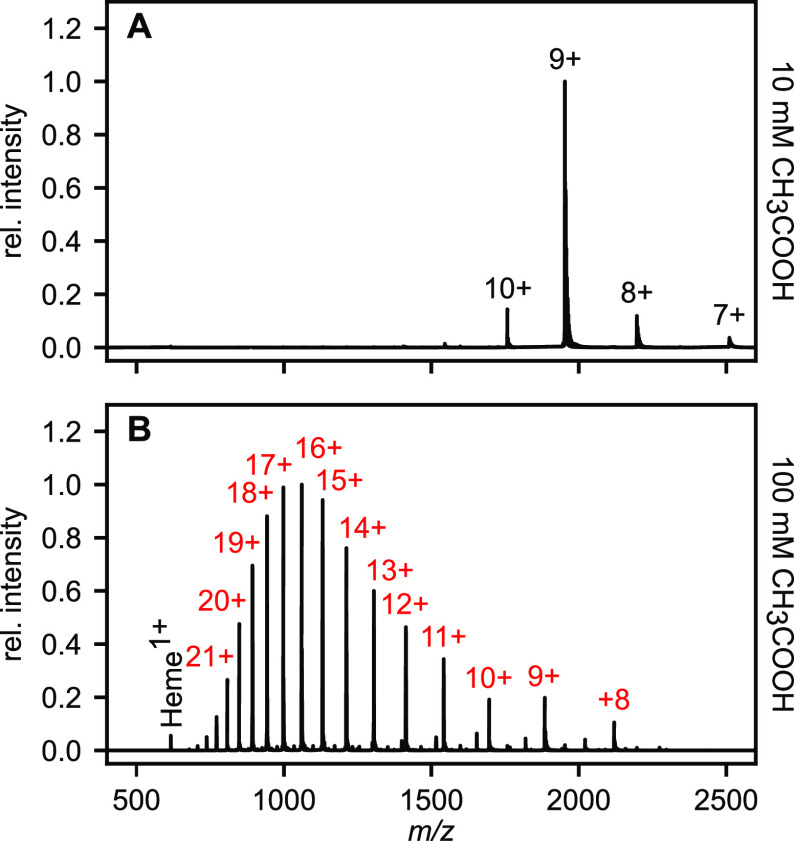
Native mass spectrometric analysis of myoglobin (10 μM) in 5 mM ammonium acetate. At 10 mM acetic acid (A) native holomyoglobin could be observed with charge states +7 to +10 (black). Under denaturing conditions at 100 mM acetic acid (B) unfolded apomyoglobin was observed with a broad charge distribution ranging from +8 to +27 (red). Released, singly charged heme could also be detected under denaturing conditions.

Nano-ESI is often used for native ionization of proteins or protein complexes. To assess the potential of the nano-ESI source for native-MS applications, the well-studied protein myoglobin was analyzed at different pH conditions (Figure 4). In ammonium acetate, myoglobin can be detected in complex with its native heme ligand with low charge states ranging from +7 to +10 and the main species carrying +9 charges (Figure 4A). When increasing the acid concentration to 100 mM, signals for released heme as well as apomyoglobin were detected with a broad charge distribution ranging from +8 to +27 (Figure 4B), indicating an extended, unfolded conformation of apomyoglobin.

## Conclusion

We devised a cost-effective 3D-printable open-source upgrade for Bruker instruments that can be used to achieve stable offline nanoelectrospray ionization. The source adapter is a simple add-on that bolts directly to the common Bruker ionBooster electrospray ionization source. All nonprinted parts can be easily sourced, and usage as well as assembly of the block are straightforward. The design is open for further optimization by the community to improve automation and stability and possibly upgrade the device for further applications.

## Materials and Methods

### Materials

Fibrinopeptide B (GluFib) was obtained from ABCR. Myoglobin, Fondaparinux, and Porcine gastric mucin (PGM) were obtained from Sigma-Aldrich (U.S.A.).

### Nano-ESI Capillaries

A Sutter instruments P-1000 micropipette puller was used to produce nano-ESI capillaries from 1 mm glass capillaries. The capillaries were subsequently sputter-coated with Pd/Pt. The end of the capillaries was clipped open under a binocular. In general, 5–10 μL of sample was introduced into the coated glass capillaries for offline analysis.

### Recommended Source Settings

To achieve a stable spray, we recommend starting within the following parameter ranges: end plate offset, 500 V; capillary voltage, 1200–1800 V; nebulizer (backing pressure), 0.5–1.5 bar; dry gas, 4 L/min; dry temperature, 150 °C.

### Analysis of Small Molecules

Fibrinopeptide B (GluFib) was dissolved at 10 μM in H_2_O/MeOH (1:1) and analyzed on a Bruker amaZon speed ETD at an emitter voltage of 1.5 kV with a mass range of *m*/*z* 200–2000. Fondaparinux (FPX) was dissolved in H_2_O/MeOH (1:1) containing 10 mM ammonium acetate. The sample was analyzed on a Bruker timsTOF Pro in negative mode at an emitter voltage of 1.4 kV with a mass range of *m*/*z* 50–3000.

### *O*-Glycan Analysis

*O*-Glycans were released from porcine gastric mucin by reductive β-elimination.^14^ Briefly, samples were incubated with 0.5 M NaBH_4_ and 50 mM NaOH (1.6 g/L) at 50 °C for 16 h. The reaction was quenched by acetic acid and desalted on Dowex 50WX8 cation exchange beads (Sigma-Aldrich, U.S.A.). *O*-Glycans were enriched on Hypercarb SPE cartridges (Thermo Fisher) and dried using a Speedvac. Glycan alditols were dissolved in H_2_O/MeOH (1:1) containing 50 mM ammonium acetate. The sample was analyzed in negative ion mode at an emitter voltage of 1.2 kV, and the mass range was set to *m*/*z* 50–2000.

### Native-MS of Myoglobin

Myoglobin was dissolved at 10 μM in 5 mM ammonium acetate with 10 mM acetic acid or 100 mM acetic acid. Bruker timsTOF settings were optimized for native protein analysis according to ref (15). Briefly, transfer funnel 1 RF was set to 300 V_PP_, transfer funnel 2 RF was set to 600 V_PP_, transfer multipole RF was set to 500 V_PP_, collision cell RF was set to 3000 V_PP_, collision cell transfer time was set to 130 μs, and prepulse storage time was set to 20 μs. The sample was analyzed in positive ionization mode at 1.4 kV emitter voltage and a mass range of *m*/*z* 50–4000.

## Data Availability

Printable STL files as well as a bill of materials to reproduce the nano-ESI source adapter are available on the open platform Printables: www.printables.com/model/410886-nanoesi-source-for-bruker.

## Supporting Information Available

Identified released *O*-glycan compositions from porcine gastric mucins (PDF)
