# Supplementary material for: A 3D-Printed Offline Nano-ESI Source for Bruker MS Instruments
Source: J Am Soc Mass Spectrom. 2023 Aug 21;34(10):2403–6. doi: 10.1021/jasms.3c00214 (PMC10557379; doi:10.1021/jasms.3c00214)

Supporting Information

A 3D Printed Offline Nano-ESI Source for Bruker MS Instruments

Michael Götze, Lukasz Polewski, Leïla Bechtella, Kevin Pagel*

Author affiliations:
Institut für Chemie und Biochemie, Freie Universität Berlin, 14195 Berlin, Germany
Fritz-Haber-Institut der Max-Planck-Gesellschaft, 14195 Berlin, Germany

Corresponding author:
Kevin Pagel
kevin.pagel@fu-berlin.de

Table S1: O-glycan compositions identified from a single analysis of released O-glycans from porcine gastric mucins (PGM).

The composition of each compound is shown using the Symbol Nomenclature For Glycans (SNFG), where the number of building blocks (>1) is noted inside each representation.

m/z (red not shown in Fig. 3)	MW in Da	Ion type	Adduct mass	Glycan composition	Representation
**384.15**	385.15	$[M-H]^-$	-1.01	Hex1HexNAc1	
**530.21**	531.21	$[M-H]^-$	-1.01	Hex1HexNAc1Fuc1	
**548.70**	1099.41	$[M-2H]^{2-}$	-2.02	Hex2HexNAc3Fuc1	
**566.19**	567.19	$[M+Cl]^-$	34.97	Hex1HexNAc1Fuc1	
**577.22**	1156.44	$[M-2H]^{2-}$	-2.02	Hex2HexNAc4	
**587.23**	588.23	$[M-H]^-$	-1.01	Hex1HexNAc2	
**621.73**	1245.46	$[M-2H]^{2-}$	-2.02	Sial1Hex2HexNAc3	
**623.21**	624.21	$[M+Cl]^-$	34.97	Hex1HexNAc2	
**650.24**	1302.48	$[M-2H]^{2-}$	-2.02	Hex2HexNAc4Fuc1	
**658.25**	1318.50	$[M-2H]^{2-}$	-2.02	Hex3HexNAc4	
**665.23**	1332.46	$[M-2H]^{2-}$	-2.02	Sial2Hex2HexNAc2	
**667.18**	668.18	$[M-H]^-$	-1.01	Hex1HexNAc2S1	
**675.24**	676.24	$[M-H]^-$	-1.01	Sial1Hex1HexNAc1	
**702.76**	1407.52	$[M-2H]^{2-}$	-2.02	Hex3HexNAc3Fuc2	
**723.27**	1448.54	$[M-2H]^{2-}$	-2.02	Hex2HexNAc4Fuc2	
**731.28**	1464.55	$[M-2H]^{2-}$	-2.02	Hex3HexNAc4Fuc1	
**733.29**	734.29	$[M-H]^-$	-1.01	Hex1HexNAc2Fuc1	
**749.28**	750.28	$[M-H]^-$	-1.01	Hex2HexNAc2	
**759.79**	1521.57	$[M-2H]^{2-}$	-2.02	Hex3HexNAc5	
**769.27**	770.27	$[M+Cl]^-$	34.97	Hex1HexNAc2Fuc1	
**790.31**	791.31	$[M-H]^-$	-1.01	Hex1HexNAc3	
**804.30**	1610.60	$[M-2H]^{2-}$	-2.02	Hex3HexNAc4Fuc2	
**813.24**	814.24	$[M-H]^-$	-1.01	Hex1HexNAc2Fuc1S1	
**821.30**	822.30	$[M-H]^-$	-1.01	Sial1Hex1HexNAc1Fuc1	
**826.29**	827.29	$[M+Cl]^-$	34.97	Hex1HexNAc3	
**832.82**	1667.63	$[M-2H]^{2-}$	-2.02	Hex3HexNAc5Fuc1	
**861.33**	1724.66	$[M-2H]^{2-}$	-2.02	Hex3HexNAc6	
**878.32**	879.32	$[M-H]^-$	-1.01	Sial1Hex1HexNAc2	
**895.34**	896.34	$[M-H]^-$	-1.01	Hex2HexNAc2Fuc1	
**936.37**	937.37	$[M-H]^-$	-1.01	Hex1HexNAc3Fuc1	
**952.36**	953.36	$[M-H]^-$	-1.01	Hex2HexNAc3	
**975.30**	976.30	$[M-H]^-$	-1.01	Hex2HexNAc2Fuc1S1	
**988.34**	989.34	$[M+Cl]^-$	34.97	Hex2HexNAc3	
**993.39**	994.39	$[M-H]^-$	-1.01	Hex1HexNAc4	
**1040.38**	1041.38	$[M-H]^-$	-1.01	Sial1Hex2HexNAc2	
**1041.40**	1042.40	$[M-H]^-$	-1.01	Hex2HexNAc2Fuc2	
**1098.42**	1099.42	$[M-H]^-$	-1.01	Hex2HexNAc3Fuc1	
**1134.40**	1135.40	$[M+Cl]^-$	34.97	Hex2HexNAc3Fuc1	
**1155.44**	1156.44	$[M-H]^-$	-1.01	Hex2HexNAc4	
**1178.38**	1179.38	$[M-H]^-$	-1.01	Hex2HexNAc3Fuc1S1	
**1191.42**	1192.42	$[M+Cl]^-$	34.97	Hex2HexNAc4	
**1301.5**	1302.50	$[M-H]^-$	-1.01	Hex2HexNAc4Fuc1	

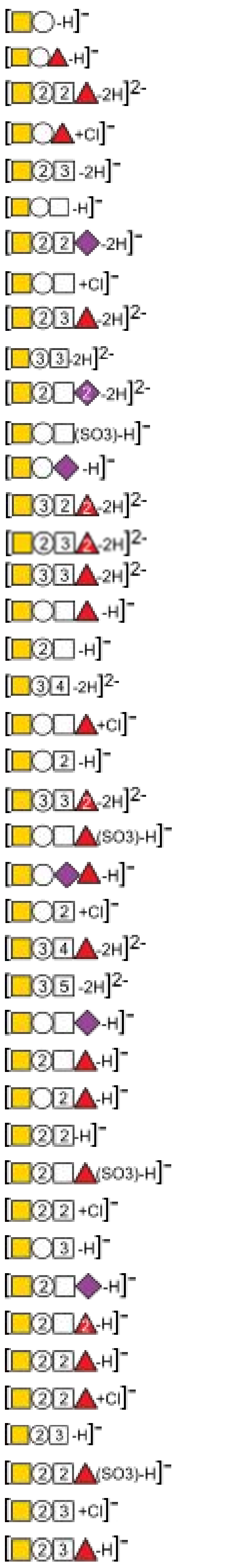

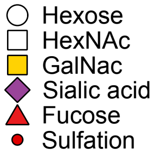

Supplement: Supplementary file 1 — js3c00214_si_001.pdf [file js3c00214_si_001.pdf]
